# *Candida albicans* cell surface superoxide dismutases degrade host-derived reactive oxygen species to escape innate immune surveillance

**DOI:** 10.1111/j.1365-2958.2008.06528.x

**Published:** 2009-01

**Authors:** Ingrid E Frohner, Christelle Bourgeois, Kristina Yatsyk, Olivia Majer, Karl Kuchler

**Affiliations:** Medical University Vienna, Christian Doppler Laboratory for Infection Biology, Max F. Perutz Laboratories, Campus Vienna BiocenterA-1030 Vienna, Austria

## Abstract

Mammalian innate immune cells produce reactive oxygen species (ROS) in the oxidative burst reaction to destroy invading microbial pathogens. Using quantitative real-time ROS assays, we show here that both yeast and filamentous forms of the opportunistic human fungal pathogen *Candida albicans* trigger ROS production in primary innate immune cells such as macrophages and dendritic cells. Through a reverse genetic approach, we demonstrate that coculture of macrophages or myeloid dendritic cells with *C. albicans* cells lacking the superoxide dismutase (SOD) Sod5 leads to massive extracellular ROS accumulation *in vitro*. ROS accumulation was further increased in coculture with fungal cells devoid of both Sod4 and Sod5. Survival experiments show that *C. albicans* mutants lacking Sod5 and Sod4 exhibit a severe loss of viability in the presence of macrophages *in vitro*. The reduced viability of *sod5*Δ/Δ and *sod4*Δ/Δ*sod5*Δ/Δ mutants relative to wild type is not evident with macrophages from *gp91phox*^−/−^ mice defective in the oxidative burst activity, demonstrating a ROS-dependent killing activity of macrophages targeting fungal pathogens. These data show a physiological role for cell surface SODs in detoxifying ROS, and suggest a mechanism whereby *C. albicans*, and perhaps many other microbial pathogens, can evade host immune surveillance *in vivo*.

## Introduction

Invasive *Candida albicans* infections are life-threatening clinical conditions affecting immunosuppressed patients and those with general defects in the immune system. The mortalities associated with disseminated candidiasis can be as high as 30–40%, despite extensive antifungal therapies (Pfaller and Diekema, 2007). Host defences against fungi range from non-specific proteolytic defences to dedicated adaptive immune responses (Romani, 2004; Netea *et al*., 2008). The earliest host response to fungal pathogens, including *C. albicans*, relies on fungal recognition by innate immune cells such as dendritic cells, macrophages and neutrophils and involves pattern recognition receptors, followed by the subsequent phagocytosis and elimination of microbial pathogens (Brown and Gordon, 2005; Akira *et al*., 2006; Jouault *et al*., 2006; Taylor, 2007; Gow *et al*., 2007).

Upon interaction with pathogens, phagocytes rapidly produce reactive oxygen species (ROS), which are thought to aid killing of invading microbes (Dinauer, 1993; Morgenstern *et al*., 1997), and further activate defensive signalling pathways reviewed in Forman and Torres (2002) and Netea *et al*. (2008). ROS production is initiated through assembly and activation of nicotinamide adenine dinucleotide phosphate (NADPH) oxidase in phagocytes (Babior, 2004). This triggers the respiratory burst by generating superoxide anions (O_2_^−^) (Schrenzel *et al*., 1998), which are subsequently converted to hydrogen peroxide (H_2_O_2_), hydroxyl radical (OH°) and hypochlorous acid, the latter conversion only taking place in neutrophils.

In *C. albicans*, the Cat1 catalase has been implicated in counteracting the respiratory burst by protecting cells from killing by H_2_O_2_ stress. Cells lacking Cat1 also display attenuated virulence in an invasive mouse virulence model as reviewed in Chauhan *et al*. (2006). Furthermore, the *C. albicans* genome harbours six genes encoding putative superoxide dismutases (SOD), four of which are copper-zinc (CuZn)-dependent, namely the cytoplasmic Sod1 and the cell surface Sod4, Sod5 and Sod6; two SODs, the mitochondrial Sod2 and cytoplasmic Sod3, are manganese-dependent (Chauhan *et al*., 2006). SODs convert O_2_^−^ into molecular oxygen and hydrogen peroxide, thereby scavenging the toxic effects of O_2_^−^ and preventing higher H_2_O_2_ levels by other downstream reactions (Teixeira *et al*., 1998).

The best-studied *C. albicans* SODs with respect to their role in pathogenesis are Sod1 and Sod5, the latter being a GPI-anchored cell surface protein (Fradin *et al*., 2005). Both appear required for virulence of *C. albicans* in invasive mouse models (Hwang *et al*., 2002). Further, fungal cells lacking Sod1 are sensitive to menadione and more sensitive to killing by macrophages than a wild-type strain (Hwang *et al*., 2002). *SOD5* is upregulated under osmotic and oxidative stress conditions, as well as during yeast-to-hyphae transition (Martchenko *et al*., 2004). Moreover, transcriptional profiling indicates that *SOD5* expression is also upregulated by neutrophil contact, in presence of neutrophils and viability of a *sod5*Δ/Δ mutant is reduced relative to the wild type. Notably, both Sod4 and Sod6 are predicted GPI-anchored cell wall proteins reviewed in Richard and Plaine (2007), but their function has not been analysed. The surface location of Sod4, Sod5 and Sod6 prompted the notion that they may protect *C. albicans* against extracellular stress (Fradin *et al*., 2005; Gantner *et al*., 2005).

In this work, we demonstrate a pivotal role for *C. albicans* SODs in destroying host-derived ROS. We show that primary innate immune cells rapidly respond to fungal pathogens by mounting a protective ROS response to destroy invading cells. We exploit a reverse genetic approach to show that certain *C. albicans* SODs counteract the respiratory burst. Strikingly, we demonstrate that Sod5, and to a lesser extent Sod4, catalyses destruction of host-derived ROS. Interestingly, *sod5*Δ/Δ and *sod4*Δ/Δ*sod5*Δ/Δ*C. albicans* show decreased viability in the presence of macrophages. Thus, our data identify *SOD5* as a novel *C. albicans* gene, mediating detoxification of host-derived ROS. The results suggest a molecular mechanism whereby fungal pathogens can escape the immediate early immune response, namely the oxidative burst reaction.

## Results

### *C. albicans* yeast and hyphae forms trigger ROS in macrophages and dendritic cells

The earliest response of innate immune cells facing pathogens includes the production of ROS (DeLeo *et al*., 1999; Forman and Torres, 2002). Thus, we asked whether *C. albicans* can induce ROS in mouse bone marrow-derived macrophages (BMDMs) as well as myeloid dendritic cells (mDCs). To investigate production of total ROS, we adapted a luminol-dependent, chemiluminescence assay in the presence of horseradish peroxidase (HRP). Oxidation of luminol by ROS leads to chemiluminescence and the luminescence measured is proportional to the ROS produced in the system (Dahlgren and Karlsson, 1999).

**Fig. 1:**
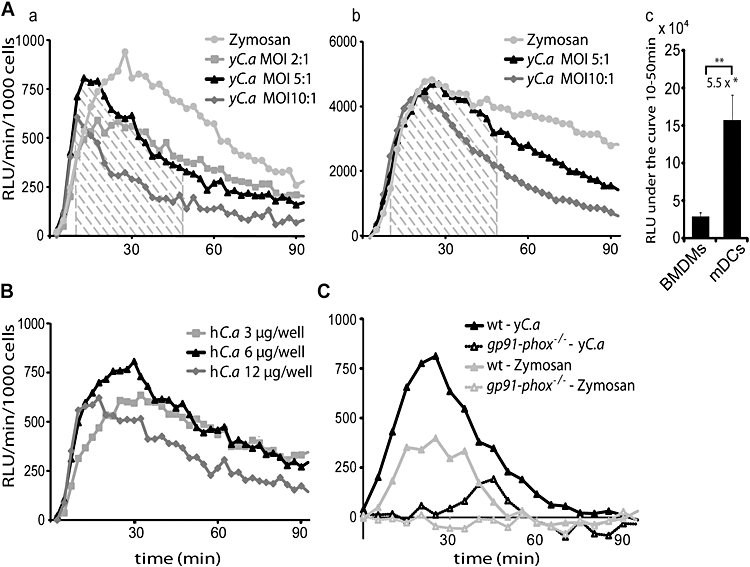
*C. albicans* induces ROS production in wild-type BMDMs and mDCs. A–C. ROS measurement by luminol-dependent chemiluminescence at 37°C in 2.5 min intervals over a 90 min period [relative luciferase units (RLU) min^−1^ per 1000 immune cells]. A. Stimulation of BMDMs (a) or mDCs (b) with yeast-form *C. albicans* (y*C.a*) at an MOI of 2:1 (equivalent to 2 μg yeast dry weight per well), 5:1 (5 μg/well) or 10:1 (10 μg/well) or with zymosan (20 μg/well). (c) Quantification of the total ROS release between 10 and 50 min (striped area) by calculating the area under the curve (MOI 5:1). The average of three independent experiments is presented. *mDCs produce 5.5 ± 0.35 times more ROS than BMDMs. **P < 0.02. B. Stimulation of BMDMs with hyphae-form *C. albicans* (h*C.a*) *at* 3 μg dry weight/well, 6 μg/well or 12 μg/well. C. Stimulation of *gp91phox*^−*/*−^ and wt BMDMs with yeast-form *C. albicans* at an MOI of 5:1 or zymosan (20 μg/well). A–C. Results of one experiment per condition are shown. Data were reproduced in at least three independent experiments. Statistical significance was calculated using a two-tailed Student's *t*-test.

In order to determine the optimal ratio of *C. albicans* to host immune cells, we first performed experiments with different multiplicities of infection (MOI). Yeast forms of the clinical isolate *C. albicans* SC5314 induced ROS in BMDMs and mDCs at an MOI ranging from 2:1 (fungi to macrophages) up to 10:1 (Fig. 1A a and b). No ROS were detected with an MOI of 20:1 and higher (data not shown). The optimal ROS response by BMDMs and mDCs was observed with a 5:1 MOI (Fig. 1A). Notably, the oxidative burst of mDCs is more than five times higher than that of BMDMs (Fig. 1A c). Zymosan, a crude cell wall preparation from *Saccharomyces cerevisiae*, served as positive control in all experiments (Gantner *et al*., 2003). Mature hyphal forms of *C. albicans* (up to 12 μg per well dry weight equivalent) also induced ROS in BMDMs (Fig. 1B). To determine whether ROS are produced by immune cells or fungi, we used BMDMs differentiated from *gp91phox*^−*/*−^ mice lacking an essential NADPH subunit required for ROS production. As expected, no ROS production was observed when *gp91phox*^−*/*−^ BMDMs were incubated with zymosan. A substantially blunted signal was detected when *C. albicans* interacted with *gp91phox*^−*/*−^ BMDMs (Fig. 1C). Thus, these data demonstrate that both yeast and hyphal forms of *C. albicans* can trigger ROS production in BMDMs as well as mDCs. Importantly, ROS detected by the assays is mainly derived from mammalian immune cells, as *gp91phox*^−*/*−^ BMDMs failed to generate ROS.

### ROS accumulate when *sod5*Δ/Δ cells infect BMDMs

Like most organisms, fungi possess various antioxidant enzymes to counteract oxidative damage, including thioredoxin, glutathione reductase, catalase, gluthathione peroxidase as well as SODs. The genome of *C. albicans* encodes six putative SODs (*SOD1–6*, reviewed in Chauhan *et al*., 2006).

To clarify which *C. albicans* SODs are involved in the response to innate immune cells, we constructed homozygous deletion strains, each lacking one of the six *C. albicans* SOD genes (*SOD1–6*) in the SN152 genetic background (Noble and Johnson, 2005). To create a *HIS1 LEU2* prototrophic control strain, we integrated the Cd*LEU2* and Cm*HIS1* cassettes at their corresponding genomic loci in the SN152 strain, yielding the strain CA-IF100, hereafter referred to as wild type throughout the text. This wild-type strain induced ROS to levels similar to the clinical isolate SC5314, suggesting that the different genetic backgrounds or auxotrophic markers did not affect ROS release (data not shown). We then tested the phenotypes of mutants lacking SODs concerning intracellular stress such as menadione, which is generating intracellular superoxide radicals, and diamide, a thiol-specific oxidant that can readily oxidize reduced glutathione. We confirmed the previously reported sensitivities of *C. albicans* strains lacking *SOD1* and *SOD2* to menadione, as well as the resistance to diamide, on SD media (Hwang *et al*., 2002; 2003) (data not shown). Importantly, the absence of extracellular SODs failed to show any sensitivity or resistance to any of the drugs causing intracellular oxidative stress, implying a putative function in extracelluar ROS detoxification.

**Fig. 2:**
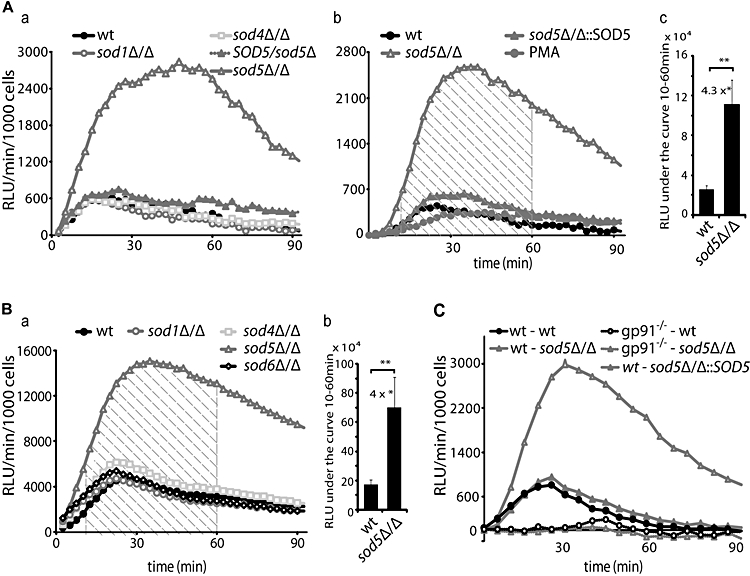
ROS accumulate when BMDMs or mDCs, but not *gp91phox*^−*/*−^ BMDMs, are infected with *sod5*Δ/Δ cells. A–C. ROS measurement by luminol-dependent chemiluminescence at 37°C in 2.5 min intervals over a 90 min period [relative luciferase units (RLU) min^−1^ per 1000 immune cells]. A. (a) Stimulation of BMDMs with either the wild type (CA-IF100) strain or the *sod1*Δ/Δ (CA-IF003), *sod4*Δ/Δ (CA-IF015), *sod5*Δ/Δ (CA-IF019) mutant strains or *sod5*Δ/*SOD5* heterozygous strain (CA-IF017) (MOI 5:1). (b) Stimulation of BMDMs with the *sod5*Δ/Δ::*SOD5* revertant (CA-IF027) (MOI 5:1) or PMA (10 nM). (c) Quantification of the total ROS release between 10 and 60 min (striped area) by calculating the area under the curve (MOI 5:1). The average of three independent experiments is presented. *Infection with *sod5*Δ/Δ yields 4.3 ± 0.68 times more ROS than with wild-type C. albicans.**P < 0.02. B. (a) Stimulation of mDCs with either the wild type (CA-IF100) strain, or the *sod1*Δ/Δ (CA-IF003), *sod4*Δ/Δ (CA-IF015), *sod6*Δ/Δ (CA-IF023) or *sod5*Δ/Δ mutant strains. (b) Quantification of the total ROS release between 10 and 60 min (striped area) by calculating the area under the curve. The average of three independent experiments is presented. *Infection with *sod5*Δ/Δ yields 4 ± 0.64 times more ROS than with wild type cells. **P < 0.05. C. Stimulation of *gp91phox*^−*/*−^ or wild-type BMDMs with either the wild-type (CA-IF100) strain, the *sod5*Δ/Δ (CA-IF019) mutant strain or *sod5*Δ/Δ::*SOD5* re-integrant (CA-IF027). A–C. Results of one experiment per condition are shown. Data were reproduced in at least three independent experiments. Statistical significances were calculated using a two-tailed Student's *t*-test.

Next, we tested phenotypes of cells lacking various SODs concerning the activation of ROS production in macrophages or dendritic cells using the luminol assay. The interaction of primary BMDMs with *C. albicans sod1*Δ/Δ or *sod4*Δ/Δ strains did not show any significant changes in ROS levels over a period of 90 min when compared with the wild-type strain CA-IF100 (Fig. 2A a). Similarly, the *sod2*Δ/Δ, *sod3*Δ/Δ and *sod6*Δ/Δ homozygous deletion strains did not show any different ROS production (data not shown). By contrast, ROS accumulated more than fourfold when BMDMs were infected with the *sod5*Δ/Δ deletion strain CA-IF019, but not with the *sod5*Δ/*SOD5* heterozygous strain (Fig. 2A a–c). As a control, we also re-integrated a functional *SOD5* gene into the corresponding genomic locus, *sod5*Δ/Δ::*SOD5* to construct the revertant strain CA-IF070. As expected, ROS levels induced by this strain were similar to those elicited by the wild-type strain. The phorbol ester PMA, a potent ROS inducer, was used as a positive control (Fig. 2A b). Similar results were obtained for ROS induction by mutant and wild-type strain using primary mDCs (Fig. 2B a and b). Furthermore, no ROS accumulation was observed in BMDMs derived from *gp91phox*^−*/*−^ mice infected with *sod5*Δ/Δ homozygous deletion strains and the wild-type strain, unequivocally demonstrating that ROS accumulation in BMDMs and mDCs requires functional *gp91phox* and the absence of Sod5 (Fig. 2C), suggesting a role for Sod5 in counteracting the oxidative burst of innate immune cells *in vitro*.

### ROS accumulation *in vitro* is due to enhanced extracellular superoxide levels

**Fig. 3:**
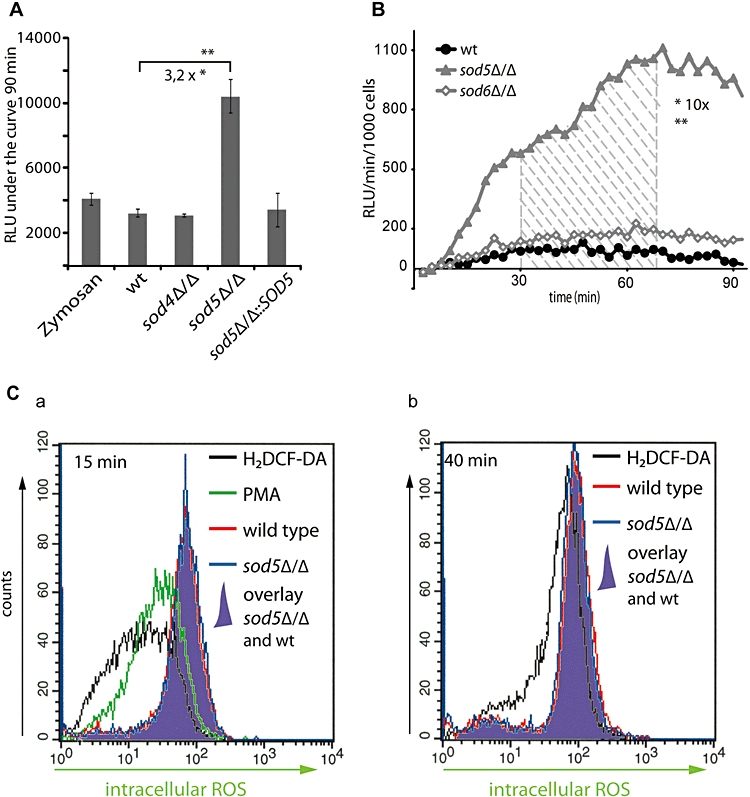
Extracellular ROS accumulate in the presence of *sod5*Δ/Δ cells. A. Superoxides measurement by lucigenin-dependent chemiluminescence at 37°C over a 90 min period [relative luciferase units (RLU) under the curve]. Stimulation of BMDMs with either the wild-type (CA-IF100) strain, or the *sod4*Δ/Δ (CA-IF015), *sod5*Δ/Δ (CA-IF019) mutant strain or the *sod5*Δ/Δ::*SOD5* revertant (CA-IF027) (MOI 5:1). *Infection with *sod5*Δ/Δ yields 3.2 ± 0.21 times more superoxides than with wild-type C. albicans**P > 0.005. B. Extracellular ROS measurement by isoluminol-dependent chemiluminescence at 37°C in 2.5 min intervals over a 90 min period [relative luciferase units (RLU) min^−1^ per 1000 cells]. Stimulation of BMDMs with either the wild-type (CA-IF100) strain or the *sod5*Δ/Δ (CA-IF019) or *sod6*Δ/Δ (CA-IF023) mutant strains (MOI 5:1). Quantification of the total ROS release between 30 and 70 min (striped area) by calculating the area under the curve. *Infection with *sod5*Δ/Δ yields 10 ± 0.5 times more extracellular ROS than with wild-type cells. **P > 0.001. C. Intracellular ROS production in response to the phorbol ester PMA, wild-type (CA-IF100) strain or *sod5*Δ/Δ (CA-IF019) mutant strain was measured by FACS analysis using H_2_DCF-DA staining of BMDMs after 15 min (a) or 40 min (b) of infection. A–C. Results of one experiment per condition are shown. All data were reproduced in at least three independent experiments. Statistical significances were calculated using a two-tailed Student's *t*-test.

The SODs are believed to destroy harmful superoxides produced by converting them first to H_2_O_2_; subsequently catalase converts H_2_O_2_ into harmless H_2_O and O_2_. We therefore hypothesized that deletion of an SOD gene should increase superoxide levels. Because the main type of ROS detected by the luminol assay is peroxide but not superoxide, we measured superoxide levels using lucigenin as a luminescence probe (Li *et al*., 1998). Superoxide accumulation in BMDMs cocultured with the wild-type strain, as well as the *sod4*Δ/Δ strain, was similar. By contrast, the *sod5*Δ/Δ mutant showed a more than threefold superoxide accumulation. As expected, superoxide accumulation was not observed in BMDMs cocultured with the functionally restored *sod5*Δ/Δ::*SOD5* strain (Fig. 3A).

The NADPH-oxidase is believed to assemble either in the plasma membrane or in membranes of phagosomes (Hampton *et al*., 1998; Kobayashi *et al*., 1998). Therefore, ROS will either be released from cells or retained inside the phagosomes. To discriminate the locations of ROS accumulation, we measured ROS using isoluminol as a luminescence probe (Lundqvist and Dahlgren, 1996), which, in contrast to luminol, is membrane-impermeable. In BMDMs, ROS accumulated about 10-fold higher in the presence of *sod5*Δ/Δ cells, when compared with macrophages coincubated with *sod6*Δ/Δ cells or the wild-type strain (Fig. 3B).

Finally, to visualize intracellular ROS production, we pre-loaded BMDMs with the non-fluorescent dye H_2_DCF-DA, which cannot cross cellular compartments after esterase cleavage. Upon oxidation by ROS, H_2_DCF-DA is converted to the fluorescent product 2′-7′-dichlorofluorescein (DCF). A limited permeability of DCF retains it preferentially at the site where it was generated (Yeung *et al*., 2005). Therefore, ROS produced in the phagosomes is not detected by H_2_DCF-DA. We then measured the generation of ROS after 15 min (Fig. 3C a) and 45 min (Fig. 3C b) using the standard 5:1 MOI of fungal cells to BMDMs and the phorbol ester PMA as a control (Fig. 3C). FACS analysis showed that intracellular ROS levels were induced at very similar levels by both *sod5*Δ/Δ and the wild-type strains (Fig. 3C, violet overlay). We conclude that Sod5 is involved in the detoxification of extracellular or phagosomal superoxides produced by BMDMs, but has no effect on the intracellular ROS levels.

### Sod4 but not Sod6 shares functional overlap with Sod5

**Fig. 4:**
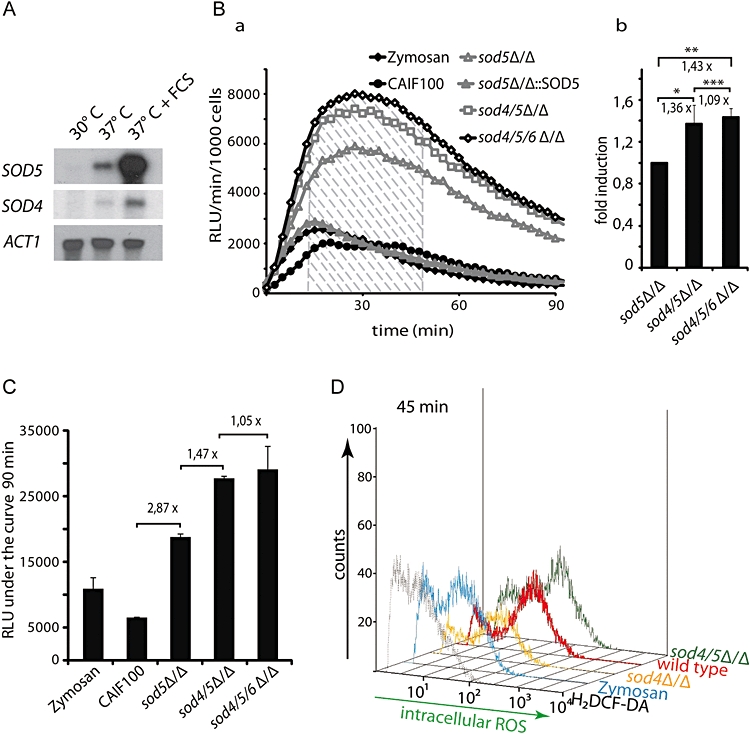
A *sod4*Δ/Δ deletion in a *sod5*Δ/Δ background boosts ROS accumulation. A. Northern analysis of *SOD4*, *SOD5* and *ACT1* mRNA. The clinical *C. albicans* SC5314 strain was grown at 30°C, 37°C and 37°C plus 10% FCS. B. ROS measurement by luminol-dependent chemiluminescence at 37°C in 2.5 min intervals over a 90 min period [relative luciferase units (RLU) min^−1^ per 1000 BMDMs]. Stimulation of BMDMs with either the wild-type (CA-IF100) strain or *sod5*Δ/Δ (CA-IF019), *sod5*Δ/Δ::*SOD5* (CA-IF027), *sod4*Δ/Δ*sod5*Δ/Δ (CA-IF039) and *sod4*Δ/Δ*sod5*Δ/Δ*sod6*Δ/Δ (CA-IF070) mutant strains (MOI 5:1) (a). Quantification of the total ROS release between 10 and 50 min (striped area) by calculating the area under the curve (MOI 5:1) and calculating the fold differences. The average of four independent experiments is presented. Infection with *sod4*Δ/Δ*sod5*Δ/Δ yields 1.36 ± 0.18 times more ROS than by a *sod5*Δ/Δ strain. *P < 0.05, *sod4*Δ/Δ*sod5*Δ/Δ*sod6*Δ/Δ triple mutant yields 1.43 ± 0.09 times more ROS than by a *sod5*Δ/Δ strain. *P < 0.02; and the *sod4*Δ/Δ*sod5*Δ/Δ*sod6*Δ/Δ triple mutant yields 1.09 ± 0.1 times more ROS than *sod4*Δ/Δ*sod5*Δ/Δ. ***P > 0.09 (b). C. Superoxides measurement by lucigenin-dependent chemiluminescence at 37°C over a 90 min period [relative luciferase units (RLU) under the curve]. Stimulation of BMDMs with either zymosan (20 μg/well), the wild-type (CA-IF100) strain, the *sod5*Δ/Δ (CA-IF019), *sod4*Δ/Δ*sod5*Δ/Δ (CA-IF039) and *sod4*Δ/Δ*sod5*Δ/Δ*sod6*Δ/Δ (CA-IF070) mutant strains (MOI 5:1). A and B. Results of one experiment per condition are shown. All data were reproduced in two independent experiments. D. Intracellular ROS production in response to wild-type (CA-IF100) strain or *sod4*Δ/Δ (CA-IF015), *sod4*Δ/Δ*sod5*Δ/Δ (CA-IF039) mutant strains (MOI 5:1) or zymosan (100 μg ml^−1^). ROS was measured by FACS analysis using H_2_DCF-DA-staining of BMDMs after 45 min of infection. A–C. Results of one experiment per condition are shown. All data were reproduced in at least three independent experiments. Statistical significances were calculated using a two-tailed Student's *t*-test.

A previous report showed that *SOD4* is upregulated in a *sod5*Δ/Δ mutant cocultured with blood cells (Fradin *et al*., 2005), suggesting that the lack of the *SOD5* gene may result in compensatory upregulation of other functionally overlapping *SOD* genes. Northern analysis demonstrated that *SOD4* mRNA levels in yeast-form *C. albicans* were lower than those of *SOD5*. However, both transcripts were strongly upregulated under conditions promoting hyphal transition, including higher temperature at 37°C or 37°C plus serum (Fig. 4A). While we failed to detect *SOD6*-specific expression via Northern analysis, we used qPCR to detect *SOD6* mRNA in the wild type, the single *sod5*Δ/Δ mutant, as well as in the *sod4*Δ/Δ*sod5*Δ/Δ double deletion strain, all of which were growing at 30°C and 37°C plus 10% FCS. The mRNA levels of *SOD6* were the same under all conditions tested (data not shown), indicating that *SOD6* is not regulated during yeast to hyphae transition or by temperature. We therefore hypothesized that *SOD4* expression may compensate at least partially for the lack of *SOD5*, while *SOD6* is unable to do so. To test this hypothesis, we generated *sod4*Δ/Δ*sod5*Δ/Δ and *sod4*Δ/Δ*sod6*Δ/Δ double mutants and a *sod4*Δ/Δ*sod5*Δ/Δ*sod6*Δ/Δ triple mutant (using the *SAT1*-flipper cassette, Reuss *et al*., 2004), and looked at ROS accumulation after infecting BMDMs. When BMDMs were infected with the *sod4*Δ/Δ*sod5*Δ/Δ double deletion strain, accumulation of ROS was slightly (1.36 times), but significantly higher than in the presence of the respective *sod5*Δ/Δ single deletion strain (Fig. 4B b). By contrast, a *sod4*Δ/Δ*sod6*Δ/Δ double mutant strain did not affect the ROS accumulation relative to single deletions or the wild-type cells (data not shown). The *sod4*Δ/Δ*sod5*Δ/Δ*sod6*Δ/Δ triple mutant slightly increased ROS accumulation when compared with the *sod4*Δ/Δ*sod5*Δ/Δ double mutant, but without a statistical significance (Fig. 4B a + b). Superoxide accumulation in BMDMs cocultured with the *sod5*Δ/Δ deletion strain was again about threefold higher than with the wild-type strain. BMDMs cocultured with the *sod4*Δ/Δ*sod5*Δ/Δ double deletion accumulated about 1.5 times more superoxides than the *sod5*Δ/Δ mutant strain. By contrast, the *sod4*Δ/Δ*sod5*Δ/Δ*sod6*Δ/Δ triple deletion strain showed no increase in superoxide accumulation relative to the *sod4*Δ/Δ*sod5*Δ/Δ double mutant (Fig. 4C).

We then measured the generation of intracellular ROS using the standard 5:1 MOI of fungal cells to BMDMs. FACS analysis showed that after 30 min intracellular ROS were induced at very similar levels by the *sod4*Δ/Δ, *sod4*Δ/Δ*sod5*Δ/Δ mutant and the wild-type strains (data not shown). Notably, after 45 min, the *sod4*Δ/Δ mutant strain exhibited less intracellular ROS than the wild-type control strain, but induced similar ROS levels as zymosan; the *sod4*Δ/Δ*sod5*Δ/Δ mutant strains induced levels of intracellular ROS similar to the wild type (Fig. 4D).

Hence, these data suggest that Sod5 and Sod4 play a major role in the clearance of ROS produced by innate immune cells. Notably, Sod4, although present at very low levels, can at least partially compensate for a loss of Sod5.

### Exogenous SOD rescues defects of cells lacking Sod4 and Sod5

Previous work indicated that a *sod5*Δ/Δ deletion strain was attenuated in a mouse model for disseminated infection, and exhibited increased susceptibility to killing by whole human blood cultures and polymorphonuclear neutrophils, but not to human monocytes or the macrophage cell line RAW264.7 (Martchenko *et al*., 2004; Fradin *et al*., 2005). Our data, as well as published virulence data, predict that cells lacking SODs should display higher susceptibilities to killing by immune cells and thus exhibit reduced viability in the presence of host cells. To examine the contribution of all CuZn-dependent SOD mutants to the defence of *C. albicans* against macrophage-derived ROS, the wild-type, *sod4*Δ/Δ, *sod5*Δ/Δ in SN152, *sod5*Δ/Δ::*SOD5*, *sod4*Δ/Δ*sod5*Δ/Δ, *sod4*Δ/Δ*sod6*Δ/Δ strains, the clinical isolate SC5314 and a new *sod5*Δ/Δ mutant generated in the genetic background of the clinical isolate SC5314 were tested for their viability in coculture with primary BMDMs using a modified ‘endpoint dilution survival’ assay as described earlier (Rocha *et al*., 2001).

**Fig. 5:**
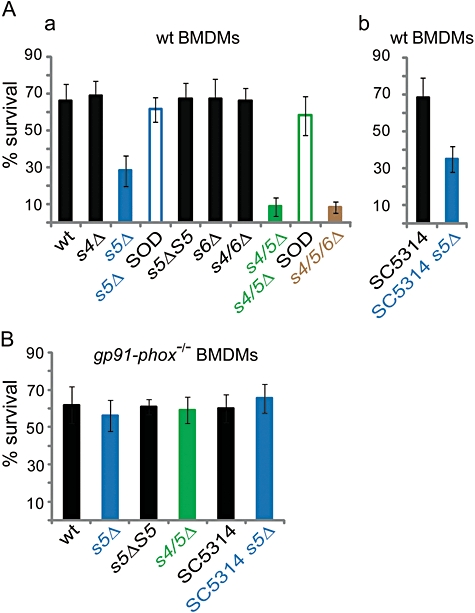
*sod5*Δ/Δ and *sod4*Δ/Δ*sod5*Δ/Δ strains are hypersensitive to killing by BMDMs. A and B. Survival of *C. albicans* and isogenic mutant cells was determined using the end-point dilution assay. Mean and standard deviation of three independent experiments are presented. A. Wild-type BMDMs in medium without (filled bars) or with 10 U commercial erythrocyte SOD (white bar) were coincubated with either wild-type (wt) *C. albicans* strain or strains lacking *SOD4* (*s4*Δ), *SOD5* (*s5*Δ blue), the restored *SOD5* (*s5*Δ*S5*), *SOD6* (*s6*Δ) or strains lacking both *SOD4* and *SOD6* (*s4/6*Δ), *SOD4* and *SOD5* (*s4*/*5*Δ green) or lacking all three *SOD4*, *SOD5* and *SOD6* (*s4/5/6* brown) (a), or with the clinical isolate SC5314 and the *sod5*Δ/Δ mutant in the SC5314 background (SC5314 *s5*Δ blue) (b) for 48 h at 37°C with 5% CO_2_. B. *gp91phox*^−*/*−^ BMDMs were infected with the wild type (wt) or strains lacking *SOD4* (*s4*Δ), *SOD5* (*s5*Δ blue), the restored *SOD5* (*s5*Δ*S5*) or strains lacking both *SOD4* and *SOD5* (*s4/5*Δ green), the clinical isolate SC5314 or the *sod5*Δ/Δ mutant in the SC5314 background (SC5314 *s5*Δ). The percentage of survival for each strain was determined as follows (colonies in absence of BMDMs versus colonies in presence of BMDMs × 100).

As shown in Fig. 5A, the quantification of the survival data of an interaction with BMDMs at the low MOI 1:1024 showed that 66.4% of the wild-type cells survived in the presence of BMDMs. Likewise, *sod4*Δ/Δ, *sod6*Δ/Δ and *sod4*Δ/Δ*sod6*Δ/Δ strains had very similar survival rates as the wild type at all BMDM dilutions. As predicted, the *sod5*Δ/Δ strain was hypersensitive to BMDM killing by almost one order of magnitude, while the *sod5*Δ/Δ::*SOD5* revertant displayed the same viability as the wild-type control (Fig. 5A). When coculturing BMDMs with the *sod4*Δ/Δ*sod5*Δ/Δ double mutant, viability was even further reduced. The *sod4*Δ/Δ*sod5*Δ/Δ*sod6*Δ/Δ triple mutant had a similar survival rate as the *sod4*Δ/Δ*sod5*Δ/Δ double mutant (Fig. 5A a), demonstrating the functional redundancy of at least Sod4 and Sod5. The increased sensitivity of *sod5*Δ/Δ and *sod4*Δ/Δ*sod5*Δ/Δ strains was observed in coincubations with BMDMs at the higher MOI of 1:4 for *sod5*Δ/Δ and 1:1 for *sod4*Δ/Δ*sod5*Δ/Δ cells respectively (data not shown). To reconfirm our findings, we also tested *sod5*Δ/Δ in the SC5314 background strain. When infected with BMDMs, *sod5*Δ/Δ SC5314 cells showed similar survival as the unrelated *sod5*Δ/Δ deletion strain CA-IF019 (Fig. 5A b).

To unequivocally demonstrate the role of SOD in mediating survival in the presence of BMDMs, we spiked survival assays with 10 U commercial bovine erythrocyte SOD enzyme. Strikingly, exogenous SOD fully rescued the viability defect to both *sod5*Δ/Δ and *sod4*Δ/Δ*sod5*Δ/Δ double mutants (Fig. 5A, white bars). Furthermore, ROS accumulation was also suppressed by the exogenous SOD activity when BMDMs were infected with strains lacking Sod5 or both Sod5 and Sod4 (data not shown). Finally, we also used *gp91phox*^−*/*−^ BMDMs to test whether the absence of ROS production can increase the survival of *sod5*Δ/Δ and *sod4*Δ/Δ*sod5*Δ/Δ strains (Fig. 5B). As expected, in the presence of *gp91phox*^−*/*−^ BMDMs, both *sod5*Δ/Δ and *sod4*Δ/Δ*sod5*Δ/Δ double mutants showed a survival comparable to the wild-type control. The same results were obtained with the independent *sod5*Δ/Δ mutant and the wild-type SC5314, respectively, in *gp91phox*^−*/*−^ BMDMs. (Fig. 5B). This proves that increased killing of the *sod5*Δ/Δ and *sod4*Δ/Δ*sod5*Δ/Δ by innate immune cells is caused by host-derived ROS. Taken together, our data demonstrate an essential role of *C. albicans* Sod5 in counteracting the host-derived immune defence as mounted through ROS to evade host immune response.

**Fig. 6:**
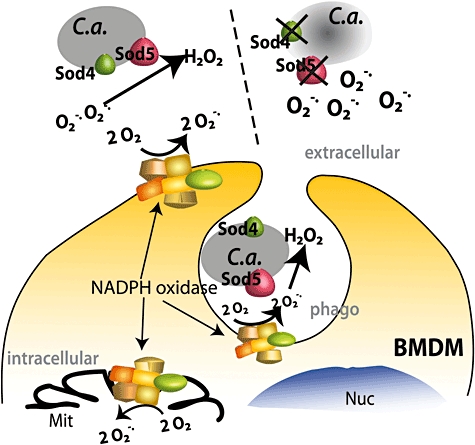
Model for Sod4 and Sod5-mediated protection against respiratory burst. Upon contact with BMDMs and mDCs, Sod4 and Sod5 anchored at the *C. albicans* (*C.a*) surface (left) degrade superoxide anions (O_2_^−^) to hydrogen peroxide (H_2_O_2_). The lack of the Sod4 and Sod5 (right) causes ROS accumulation in the medium and perhaps inside the phagosomes (phago), which results in enhanced killing of *C. albicans*. Production of mitochondrial ROS (Mit) is unaffected.

Based on our results, we propose that *C. albicans* can escape host-generated oxidative burst (Fig. 6). Adhesion, recognition and phagocytosis of fungal cells by innate immune cells trigger an immediate and rapid assembly of the ROS machinery at the cell surface or in the forming phagosomal membrane, preceding phagocytosis and persisting throughout phagosomal formation (Nauseef, 2004). Concomitantly, host temperature and adhesion may enhance *SOD4* and *SOD5* expression, followed by the elimination of extracellular and perhaps phagosomal ROS produced by host cells. In our *in vitro* assay during phagocytosis, substrate and enzyme may become trapped in the phagosomes. Hence, ROS production may also continue within the phagosomes. The SOD-mediated decay of host-derived ROS perhaps facilitates intraphagosomal survival of fungal cells, which would facilitate killing of the host cells. Taken together, these data reveal a physiological function of cell surface SODs in evading immune surveillance, thereby facilitating invasion and ultimately dissemination of fungal pathogens in the mammalian host (Fig. 6).

## Discussion

In this report, we show that yeast and hyphal forms of *C. albicans* rapidly induce ROS in primary innate immune cells such as macrophages and dendritic cells. We demonstrate that the GPI-anchored Sod5 and Sod4 enzymes act to degrade extracellular ROS produced by innate immune cells. Strikingly, *C. albicans* strains lacking SODs Sod4 and Sod5 fail to counteract the host-derived oxidative burst and are thus hyper-susceptible to killing by primary BMDMs, suggesting a physiological role of cell surface SODs in the evasion of immune surveillance.

### Yeast and hyphae forms of *C. albicans* induce ROS in BMDMs and mDCs

The ROS induction is independent of morphology as both yeast and filamentous forms of *C. albicans* trigger ROS in BMDMs (Fig. 1A). Our data agree in principle with previous studies showing ROS production upon fungal recognition (Gantner *et al*., 2005), but in contrast to this earlier report, we found that hyphae also have the capacity to trigger ROS in BMDMs (Fig. 1B a). This discrepancy may be due to differences in experimental conditions. Notably, the previous study used higher MOI than our study. In our hands, increasing MOI to similar high levels failed to trigger ROS during the interaction of both yeast and hyphal forms with BMDMs (data not shown), suggesting that higher amounts of *C. albicans* may kill or exceed the macrophage defence capacity.

We observed about 5.5 times more ROS upon interaction of *C. albicans* with mDCs when compared with BMDMs, perhaps as a consequence of higher NADPH oxidase activities in mDCs (Fig. 1A a). Consistent with this notion, similar observations were made in mDC responding to the phorbol ester PMA (Savina *et al*., 2006), one of the strongest ROS triggers known. We unequivocally demonstrate that the majority of ROS produced in response to *C. albicans* is produced through the NADPH oxidase present in immune cells, as ROS release is almost absent in *gp91phox*^−*/*−^ cells lacking a functional oxidase (Fig. 1C).

Host cells produce ROS in response to *C. albicans*, as well as fungal surface structures, although the molecular identities of ligands triggering ROS signalling remain unknown. Possible candidates include beta1–3 as well as beta 1–6 glucans (Gantner *et al*., 2003; Rubin-Bejerano *et al*., 2007). However, the use of appropriate knock-out mice may allow to answer which pattern recognition receptors contribute to ROS signalling or mediate *C. albicans* uptake into host cells (Netea *et al*., 2008).

### *C. albicans* Sod5 degrades extracellular ROS produced by immune cells

Experiments using monocyte-derived dendritic cells from human blood show that *C. albicans* inhibit PMA-induced superoxide production. This inhibition increases with increasing numbers of *C. albicans* cells, whereas heat-killed *C. albicans* fails to do so (Donini *et al*., 2007). Based on our work, we propose that *C. albicans* actively counteracts the oxidative burst of immune cells by expressing and inducing expression of cell surface SODs, which may therefore be considered fungal defence genes (Fig. 6). The GPI-anchored Sod5 and Sod4, as well as Sod6, have only been described in *C. albicans* so far. However, BlastP or tBlastN analysis identified at least one coding sequence potentially encoding putative GPI-anchored homologous of *SOD4*, *SOD5* or *SOD6* in other fungal pathogens, including *Candida dubliniensis*, *Candida tropicalis*, *Candida parapsilosis*, *Candida guilliermondi*, *Debraryomyces hanseii* and *Lodderomyces elongisporus* (data not shown). Hence, these pathogens may rely on similar mechanisms to counteract host-derived oxidative stress. Infecting BMDMs and mDCs with *C. albicans* mutants lacking putative Sod enzymes shows that Sod5 can degrade extracellular and maybe phagosomal superoxides, but not intracellular, superoxides produced by BMDMs and mDCs (Fig. 3 and 4). Thus, to the best of our knowledge, this is the first report that a fungal cell surface SOD degrades extracellular ROS released by host cells.

The ability of *C. albicans* to destroy ROS *in vitro* may explain why despite its cytotoxic potential, macrophages are poor in killing *C. albicans.* Notably, even if only a small fraction of fungal cells survive and escape phagosomal killing to grow within the host, the subsequent filament formation will physically destroy the host cell (Mansour and Levitz, 2002). Furthermore, we and others (Martchenko *et al*., 2004) show that elevated temperature, yeast-to-hyphae transition (Fig. 4A), as well as conditions mimicking the phagosome environment, strongly induce *SOD5*. Similarly, contact with neutrophils strongly activates *SOD5* transcription (Fradin *et al*., 2005). Thus, *SOD5* upregulation is perhaps part of the mechanism whereby the pathogen defence machinery responds to adverse host conditions.

The upregulation of *SOD4* in a *sod5*Δ/Δ deletion strain (Fradin *et al*., 2005) partially compensates for the loss of Sod5, providing redundant function. Indeed, we show that the putative extracellular Sod4 also contributes to ROS degradation, although at much lower capacity (Fig. 4B and C). Interestingly, microarray data and our own preliminary results (data not shown) suggest that Sod4 is also upregulated upon the transition from the white form to the opaque form of *C. albicans* (Lan *et al*., 2002), implying that Sod4 may also play a prominent role in ROS degradation in the opaque form. Opaque phase *C. albicans* cells, for instance, are better colonizers of the skin and are also believed to colonize the anaerobic gastrointestinal tract. Hence, Sod4 could play a more prominent role in the gastrointestinal tract or in skin infections. By contrast, white cells are more prevalent in bloodstream infections (Kvaal *et al*., 1999; Dumitru *et al*., 2007; Ramirez-Zavala *et al*., 2008), providing selective advantages for the survival of opaque versus white cells in different host niches.

Notably, we were unable to detect a role in ROS decay for Sod6, the third *C. albicans* SOD predicted to reside at the cell surface. We detected *SOD6* mRNA in the wild type, the *sod5* single as well as the *sod4 sod5* double deletion strains in YPD 30°C or YPD + FCS 37°C (data not shown). Furthermore, removal of *SOD6* in the *sod4*Δ/Δ or the *sod4*Δ/Δ*sod5*Δ/Δ deletion strains does not play a role in ROS degradation *in vitro*, at least in the interaction with primary BMDMs. *In vivo* experiments using animal models might provide more details as to a possible protective function of *SOD6*. Moreover, a surface localization of Sod6 has not been demonstrated or published. Therefore, the possibility remains that Sod6 may also reside in another cellular compartment, explaining the lack of ROS recognition during host interaction.

### Cells lacking Sod4 and Sod5 are hypersensitive to killing by host ROS

*Candida albicans* strains lacking the *SOD5* gene display attenuated virulence in mice *in vivo* (Martchenko *et al*., 2004), and contribute to a better survival of *C. albicans* in neutrophils (Fradin *et al*., 2005). This is in agreement with our *in vitro* survival experiments, showing that *sod5*Δ/Δ mutant cells in two independent genetic backgrounds show strongly reduced survival in BMDMs when compared with the wild-type control strain, and the genomically restored *SOD5* revertant (Fig. 5A). However, our results are not in agreement with a previous study, reporting similar survival degrees of the *sod5* mutant when compared with the wild-type strain (Martchenko *et al*., 2004). However, the previous study used the macrophage cell line RAW264.7, whereas we exploited primary macrophages, which are likely to display a pathogen response reminiscent of the normal host situation. Hence, the RAW264.7 tumour cells might very well display a different signalling response to *C. albicans* than unstimulated primary BMDMs. Further, the ‘immortalized’ tumour RAW264.7 cells in question stem from different progenitors than our BMDMs, as they were isolated from ascites and not from bone marrow. Interestingly, a recent report indicates that *C. albicans* is more susceptible when applying the end-point dilution survival assays with RAW264.7 cells than with BMDMs (Marcil *et al*., 2008).

Cells lacking Sod4 and Sod5 show significantly decreased survival when compared with the single *sod5*Δ/Δ mutant, confirming the importance of Sod4 activity and the functional redundancy with Sod5. Further, complementing the defect with commercial SOD from bovine erythrocytes restores the survival of mutant strains to almost wild-type levels. (Fig. 5A, white bars). Moreover, wild type, *sod5*Δ/Δ and *sod4*Δ/Δ*sod5*Δ/Δ strains are all equally sensitive to killing by *gp91phox*^−*/*−^ BMDMs defective in ROS release. The remaining 30–40% killing efficiency of *gp91phox*^−*/*−^ macrophages, as well as the 30–40% killing of wild-type *C. albicans* strains by wild-type BMDMs, may be independent of the oxidative burst and stem from other host defence mechanisms such as acidification of the phagolysosomes (Watanabe *et al*., 1991).

Our results recall previous findings showing that extracellular CuZn SODs of bacteria, for example SodC of *Mycobacterium tuberculosis* and the periplasmic SodC of *Salmonella typhimurium* confer improved survival in macrophages by degrading extracellular superoxides (De Groote *et al*., 1997; Piddington *et al*., 2001). Our current working model suggests that *C. albicans* can eliminate ROS produced in the extracellular space of the macrophages and dendritic cells, including ROS produced during phagosome formation within immune cells (Fig. 6).

Taken together, this work suggests that pathogens able to develop high oxidative stress tolerance are also more resistant to killing by immune cells. Therefore, scavenging ROS produced by the NADPH oxidase reaction through surface SODs may represent a physiological mechanism driving virulence, invasion and efficient survival in the host. The work also suggests a general mechanism whereby *C. albicans* and other fungal pathogens evade the host immune response and surveillance. Hence, inhibiting or blocking the extracellular SOD enzymes of *C. albicans* may be a novel therapeutic approach to combat systemic fungal disease. For instance, specific inhibitors of SODs may prove useful novel drugs to be used alone or in combination with existing antifungals to interfere or block dissemination of fungal pathogens *in vivo*.

## Experimental procedures

### Reagents, media and growth conditions

Luminol, Lucigenin, Isoluminol, HRP Type VI, PMA, SOD from bovine erythrocytes and zymosan were obtained from Sigma (St Louis, MO). FCS, HBSS, H_2_DCF-DA were from Invitrogen Molecular Probes (Oregon). DMEM was purchased from PAA (Vienna, Austria), anti-mouse antibodies CD16/CD32, CD11b-FITC, CD11c-APC, F4/80-PE-Cy5 were obtained from BD Bioscience (Mountain View, CA). Rich medium (YPD) and synthetic complete were prepared essentially as described (Kaiser *et al*., 1994). BMDM media are composed of DMEM, 10% heat-inactivated FCS, 20% l-conditioned medium. mDC media are composed of DMEM, 10% heat-inactivated FCS, 10% X-conditioned medium. *C. albicans* strains were grown at 30°C in YPD medium overnight, diluted to an OD_600_ = 0.2 the next morning, grown to the logarithmic growth phase and used for the experiment unless indicated otherwise. For the preparation of mature filaments, an overnight culture of *C. albicans* was diluted 1:10 in YPD + 10% FCS and grown at 37°C for 3–4 h. For experiments requiring stimulation of macrophages with filaments, an aliquot of each culture was pelleted and the dry weight was determined by routine procedures. Aliquots of cultures equalling the indicated dry weights of yeast or filaments were used for experiments. Typically, 4 × 10^4^ yeast cells correspond to 1 μg dry weight.

### Fungal strains and construction of *C. albicans* deletion mutants

*Candida albicans* strains, primers and plasmids used in this study are listed in Tables S1–S3 respectively. The laboratory strain SN152 served as wild-type parental strain to construct single deletion strains (*SOD1* to *SOD6*) using the method described elsewhere (Noble and Johnson, 2005). SN152 is a leucine, histidine, arginine auxotroph derivative of the clinical isolate SC5314 (Gillum *et al*., 1984). The *sod5* deletion was also generated in the SC5314 background. Multiple gene deletion mutants, as well as the *sod5*Δ/Δ in the SC5314 background, were created using the recyclable ‘*SAT1*-flipping’ method (Reuss *et al*., 2004). Transformation was achieved by electroporation (Reuss *et al*., 2004). For all strains used in this study, correct genomic integration was verified by PCR and Southern blotting.

### Mouse strains and cell culture of innate immune cells

The 7- to 9-week-old C57BL/6 wild-type mice were used for preparation of BMDMs and mDCs. Frozen bone marrow of 6- to 8-week-old *gp91phox*^−*/*−^ C57BL/6 mice was kindly provided by Kristina Erikson (George-Chandy *et al*., 2008). Bone marrow was collected from mouse femurs, treated with red blood lysis buffer (8.29 g l^−1^ NH4CL, 1 g l^−1^ KHCO3, 0.0372 g l^−1^ EDTA, pH 7.2–7.4) and re-suspended either in macrophage media to induce differentiation into BMDMs or in mDC media to prepare mDC according to previously described methods (Hume and Gordon, 1983; Inaba *et al*., 1992). After 3 days in culture, fresh medium was added. mDCs were used after 7–8 days in culture. After 7 days, BMDMs cultures were split 1:3 and further cultured up to day 10. BMDMs were used between day 10 and day 13 of differentiation. Cell surface markers of the mDCs and BMDMs cell preparation were assessed by flow cytometry using a panel of marker antibodies. mDCs preparations were negative for F4/80, a macrophage marker, positive for CD11b, and at least 50–60% of the cells were CD11c+. In BMDMs cultures, 95% of the cells expressed CD11b and F4/80 markers.

### ROS assays

For the detection of total, extracellular and intracellular ROS, chemiluminescense assays were performed using electron acceptors with various characteristics; luminol- (reacts weakly with O_2_^−^, strongly with other ROS like H_2_O_2_, HRP-dependent), isoluminol- (extracellular O_2_^−^, HRP dependent) and lucigenin- (O_2_^−^) enhanced chemiluminescence assays were performed as described before (Dahlgren and Karlsson, 1999). Briefly, BMDMs were suspended in culture medium at a density of 4 × 10^5^ cells ml^−1^ and kept warm at 37°C in a water bath for a maximum of 30 min. And 100 μl aliquots of cell suspension were distributed in a 96-well luminometer plate (Nunc, Roskilde, Denmark); 50 μl HBSS medium containing PMA (10 nM) or zymosan (100 μg ml^−1^) as positive controls and *C. albicans* mutants at the indicated cell numbers were added. Immediately after adding stimuli, 50 μl HBSS containing either 200 μM luminol or 600 μM Isoluminol and 16 U HRP, or 400 μM lucigenin were distributed into each well. Chemiluminescence was measured at 2.5 min intervals at 37°C with a multiplate reader Wallac VictorV^3^ (PerkinElmer). Data are expressed as relative luciferase units min^−1^ per 1000 BMDM cells over time, or as total relative luciferase units under curve within 90 min. Area under the curve was calculated using the trapezoidal method. Statistical significances were calculated using two-tailed Student's *t*-test from three wells per condition or from data of three independent experiments.

Intracellular ROS was measured using H_2_DCFA-DA dye to determine hydrogen peroxide production. BMDMs were suspended in HBSS at 5 × 10^6^ cells ml^−1^ approximately 30 min before measurements. Just prior to the experiment, cells were loaded with 5 μM H_2_DCF-DA in HBSS for 20 min at room temperature in the dark, and pelleted at 300 *g* for 7 min at room temperature. After washing with PBS, cells were carefully re-suspended in HBSS at a density of 5 × 10^6^ cells ml^−1^. Aliquots of 5 × 10^5^ cells were stimulated with different agents in HBSS. *C. albicans* (MOI 5:1) zymosan (1 mg ml^−1^), PMA (200 nM) and incubated for 15–45 min at 37°C. After an additional washing step, cells were re-suspended in 400 μl PBS, 0.1% BSA on ice, followed by FACS analysis with FL1-H.

### RNA extraction and Northern analysis

Total yeast RNA was isolated by the hot phenol method and quantified exactly as described elsewhere (Kren *et al*., 2003). About 15 μg of total RNA per sample was separated in a 1.4% agarose gel and transferred to nylon membranes (Amersham, Buckinghamshire, UK). Northern blots were hybridized with PCR-amplified probes, which were ^32^P-dCTP-radiolabelled by using a MegaPrime labelling kit (Amersham) using conditions recommended by the manufacturer. Hybridization with purified probes was performed exactly as previously described (Kren *et al*., 2003). Membranes were washed three times in 2× SSC-1% SDS and three times in 1× SSC-1% SDS at 65°C, and then exposed to X-ray films at −70°C. DNA probes for Northern blots were PCR-amplified from genomic DNA using primers listed in Table S1.

### End-point dilution survival assays

End-point dilution survival assays were performed as described previously (Rocha *et al*., 2001) with the following modifications. BMDMs were seeded 1 day before the experiment at 1 × 10^5^ cells per well in every second column of flat-bottom 96-well plates (Greiner, Longwood, Florida) in BMDM medium. Next day, cells were washed twice with PBS and 100 μl DMEM without phenol red containing 10% FCS. Overnight cultures of *C. albicans* cells were washed in PBS, and re-suspended at 2 × 10^6^ cells ml^−1^ DMEM without phenol red but with 10% FCS. Aliquots of 50 μl cell suspensions were added to the first two columns, and serial fourfold dilutions of *C. albicans* suspensions were placed in subsequent columns. Plates were spun at 500 *g* for 1 min, followed by incubation at 37°C and 5% CO_2_ for 48 h. Yeast colonies were stained in the 96-well plate with Cristal violet, using a 0.2% solution in 20% MeOH exactly as described previously (Stockinger *et al*., 2002). Viable colonies were counted and compared with equivalent dilutions in wells with macrophages. An assay setup of four to eight plates per day was defined as one experiment. At least three independent experiments were performed per condition. Colonies from a total of at least 25 wells per condition were used to quantify viability data.
