# Supplementary material for: Candida albicans cell surface superoxide dismutases degrade host-derived reactive oxygen species to escape innate immune surveillance
Source: Mol Microbiol. 2009 Jan;71(1):240–52. doi: 10.1111/j.1365-2958.2008.06528.x (PMC2713856; doi:10.1111/j.1365-2958.2008.06528.x)
Supplement: Supplementary file 1 [file mmi0071-0240-SD1.pdf]

Table S1: Oligonucleotide primers used in this study

**Primers to generate deletion cassettes – direction 5' → 3'**	
M5	ccgctgctaggcgcgccgtgACCAGTGTGATGGATATCTGC
M3	gcagggatgcggccgctgacAGCTCGGATCCACTAGTAACG
55_caSOD1s	CAAGTCCATCTAAAATGTGTTTG
53_caSOD1as	cacggcgcgcctagcagcggCATTTTTAATTATATATATGTTGATAATTGAAT
35_caSOD1s	gtcagcggccgcatccctgcTGAATAGATGAGCCAAGATTGC
33_caSOD1as	ATGTGGGCATTATATTTGAACC
55_caSOD2s	gtgtgatttctcacacaccaatc
53_caSOD2as	cacggcgcgcctagcagcggCATTGTTAATTATAGTACAATTGTCTTTAAT
35_caSOD2s	gtcagcggccgcatccctgcTAAGTTACTGGACAAAAGTCAAGTACA
33_caSOD2as	GAGTTCTAAACAATGGTACTTATCCTAC
55_caSOD3s	AGGGAAACTTACCATGAATGTG
53_caSOD3as	cacggcgcgcctagcagcggCATGGCGTGGTTGATAAGAG
35_caSOD3s	gtcagcggccgcatccctgcGATACTCTGCGTAACATTGTGTGTA
33_caSOD3as	CCAATTAACCCTTCGGTAGTG
55_caSOD4s	CAGCATAAACCAAATAACATTACTC
53_caSOD4as	cacggcgcgcctagcagcggCATAGTAATAGTGTGTGTGATTAAAAATC
35_caSOD4s	gtcagcggccgcatccctgcTAGATAGAGAATAACTAGAACAATCAAATG
33_caSOD4as	CTTGAAAAAATATCATTAAGTGAACG
55_caSOD5s	CACGGCTGAGAGGTCACTAC
53_caSOD5as	cacggcgcgcctagcagcggCATGATGAATGGTAAGTTAGATTG
35_caSOD5s	gtcagcggccgcatccctgcAGATGAGCCATTTTACTTATTGTG
33_caSOD5as	CATGTCTGTATAGGATAATGAAAGTG
55_caSOD6s	GCTTGGTAGTGGTGGACTAGAG
53_caSOD6as	cacggcgcgcctagcagcggCATCTTGCTGAGACGTTTAGTG
35_caSOD6s	gtcagcggccgcatccctgcTAGTTGAACATAAATACTCTCACCC
33_caSOD6as	CGATTCAGAGCTTGAGATTGAG
**Control primers for verifying genomic deletions – direction 5' → 3'**	
5_SOD1s	CATTCAAAGACAGGTTGAATACAAC
3_SOD1as	CAACAAAGTGATATTAATCGAATGAC
5_SOD2s	CCAAATAGACATAAATTTCGGTTC
3_SOD2as	TCAATCATAATGTTTATAGGACTGG
5_SOD3s	ATCTACTGGTATGAAGATTTGGTTAG
3_SOD3as	CAAAGCTCCAATCAATCCAAG
5_SOD4s	AACCTCCTAAACGCAACTGC
3_SOD4as	GAACCAAGGAAGCATTGCC
5_SOD5s	CGGCAATTGATTACGACAAG
3_SOD5as	CTCACGTTTGCTTCTCGC
5_SOD6s	GAGGCATCTGTTGCTTCCAC
3_SOD6as	CGGTAGACTATTTGTCATTGGTG
Leu2as	GGAAACATTCACACAACCTGGG
Leu1s	ccggtttacttggatcttcgg
His2as	CCCATACTCCTCACACAACAATCC
His1s	gccatgagcaccataaggacg
**Primers to generate deletion cassettes with pSF2a SAT1-FLP – direction 5' → 3'**	
55_SacI_SOD4s	GAgagctcCAGCATAAACCAAATAACATTACTC
53_SOD4_NotIas	GAgcggccgcCATAGTAATAGTGTGTGTGATTAAAAATC

35_ApaI_SOD4s	GAgggcccTAGATAGAGAATAACTAGAACAATCAAATG
33_SOD4_KpnIas	GAggtaccCTTGAAAAAATATCATTAAGTGAACG
55_SacI_SOD5s	GAgagctcCACGGCTGAGAGGTCACTAC
53_SOD5_NotIas	GAgcggccgcCATGATGAATGGTAAGTTAGATTG
35_ApaI_SOD5s	GAgggcccAGATGAGCCATTTTACTTATTGTG
33_SOD5_KpnIas	GAggtaccCATGTCTGTATAGGATAATGAAAGTG
55_SacI_SOD6s	GAgagctcGCTTGGTAGTGGTGGACTAGAG
53_SOD6_NotIas	GAgcggccgcCATCTTGCTGAGACGTTTAGTG
35_ApaI_SOD6s	GAgggcccTAGTTGAACATAAATACTCTCACCC
33_SOD6_KpnIas	GAggtaccCGATTCAGAGCTTGAGATTGAG
SAT108as	CTCCATCACCCAGTTTAGTTGTACC
SAT101s	CTCAAGTCTCGAACGAAACAG
**Primers for genomic reintegration cassettes at original loci – direction 5' → 3'**	
33_SOD5_PvuIs	GAcgatcgCATGTCTGTATAGGATAATGAAAGTG
SOD5_ct_Notas	GAgcggccgcATTTTATTTTTCTTTTTTAAATCAAGGC
**Primers for amplifying probes for Northern analysis – direction 5' → 3'**	
Sod5_49s	GATGCACCAATCTCAACTG
Sod5_676as	CAGCAATGACACCAACTAC
Sod4_9s	CTTGTCTATTATTTCAATTGTTGC
Sod4_699as	CTAAATTAAAGCAGCAACAACAC
ACT1_s	ATGGACGGTGGTATGTTTTAGT
ACT1_as	CAGAAGATTGAGAAGAAGTTTGC

Table S2: Plasmids used in this study

Plasmids	Relevant inserts and cloning sites	References
pSFS2a-SAT1-FLP		Reuss et al, 2004
pSN40		Noble & Johnson, 2005
pSN51		Noble & Johnson, 2005
pSFS2a-SOD4	*SacI*5'SOD4*NotI*-SAT1-FLP-*ApaI*3'SOD4*KpnI*	This study
pSFS2a-SOD5	*SacI*5'SOD5*NotI*-SAT1-FLP-*ApaI*3'SOD5*KpnI*	This study
pSFS2a-SOD6	*SacI*5'SOD6ct*NotI*-SAT1-FLP-*ApaI*3'SOD6*KpnI*	This study
pSFS2a-SOD5rev	*SacI*5'SOD5ct*NotI*-SAT1-FLP-*ApaI*3'SOD5*PvuI*	This study

Table S3: Fungal strains used in this study

Strains	Short Names	Genotypes	References
SC5314			Gillum, et al, 1984
SN152	*arg4Δ/Δ leu2Δ/Δ his1Δ/Δ*	*arg4Δ/arg4Δ, leu2Δ/leu2Δ, his1Δ/his1Δ, URA3/ura3Δ*	Noble & Johnson, 2005
CA-IF100	*arg4Δ/Δ LEU2 HIS1*	*arg4Δ/arg4Δ, leu2Δ/leu2Δ::cmLEU2, his1Δ/his1Δ::cdHIS1, URA3/ura3Δ,*	This study
CA-IF001	*sod1Δ*	SN152, *sod1Δ::cmLEU2/SOD1*	This study
CA-IF003	*sod1Δ/Δ*	SN152, *sod1Δ::cmLEU2/sod1Δ::CdHIS1*	This study
CA-IF005	*sod2Δ*	SN152, *sod2Δ::cmLEU2/SOD1*	This study
CA-IF007	*sod2Δ/Δ*	SN152, *sod2Δ::cmLEU2/sod2Δ::CdHIS1*	This study
CA-IF009	*sod3Δ*	SN152, *sod3Δ::cmLEU2/SOD2*	This study
CA-IF011	*sod3Δ/Δ*	SN152, *sod3Δ::cmLEU2/sod3Δ::CdHIS1*	This study
CA-IF013	*sod4Δ*	SN152, *sod4Δ::cmLEU2/SOD4*	This study
CA-IF015	*sod4Δ/Δ*	SN152, *sod4Δ::cmLEU2/sod4Δ::CdHIS1*	This study
CA-IF017	*sod5Δ*	SN152, *sod5Δ::cmLEU2/SOD5*	This study
CA-IF019	*sod5Δ/Δ*	SN152, *sod5Δ::cmLEU2/sod5Δ::CdHIS1*	This study
CA-IF025	*sod5Δ/SOD5*	SN152, *sod5Δ::cmLEU1/sod5Δ::CdHIS1::SOD5-SAT1-FLP*	This study
CA-IF027	*sod5Δ/SOD5*	SN152, *sod5Δ::cmLEU1/sod5Δ::CdHIS1::SOD5-FRT*	This study
CA-IF030	*sod5Δ/Δ sod4Δ*	SN152, *sod5Δ::cmLEU1/sod5Δ::CdHIS1 sod4Δ::SAT1*-FLP/*SOD4*	This study
CA-IF033	*sod5Δ/Δ sod4Δ*	SN152, *sod5Δ::*cm*LEU1/sod5Δ::CdHIS1 sod4Δ::*FRT/*SOD4*	This study
CA-IF036	*sod5Δ/Δ sod4Δ/Δ*	SN152, *sod5Δ::*cm*LEU1/sod5Δ::CdHIS1 sod4Δ::*FRT/*sod4Δ::SAT1*-FLP	This study
CA-IF039	*sod4/5Δ/Δ*	SN152, *sod5Δ::*cm*LEU1/sod5Δ::Cd HIS1 sod4Δ::*FRT/*sod4Δ::*FRT	This study
CA-IF021	*sod6Δ*	SN152, *sod6Δ::*cm*LEU2/SOD6*	This study
CA-IF023	*sod6Δ/Δ*	SN152, *sod6Δ::*cm*LEU1/sod6Δ::*Cd*HIS1*	This study
CA-IF043	*sod6Δ/Δ sod4Δ*	SN152, *sod6Δ::*cm*LEU1/sod6Δ::*Cd*HIS1 sod4Δ::SAT1-FLP/SOD4*	This study
CA-IF046	*sod6Δ/Δ sod4Δ*	SN152, *sod6Δ::*cm*LEU1/sod6Δ::*Cd*HIS1 sod4Δ::*FRT/*SOD4*	This study
CA-IF049	*sod6Δ/Δ sod4Δ/Δ*	SN152, *sod6Δ::*cm*LEU1/sod6Δ::*Cd*HIS1 sod4Δ::*FRT/*sod4Δ::SAT1-FLP*	This study
CA-IF051	*sod4/6Δ/Δ*	SN152, *sod6Δ::*cm*LEU*1/*sod6Δ::*Cd*HIS1 sod4Δ::*FRT/*sod4Δ::*FRT	This study
CA-IF054	*sod5Δ*	SC5314, *sod5Δ::SAT1*-FLP/*SOD5*	This study
CA-IF057	*sod5Δ*	SC5314, *sod5Δ::*FRT/*SOD5*	This study
CA-IF060	*sod5Δ/Δ*	SC5314, *sod5Δ::*FRT/ *sod5Δ::SAT1*-FLP	This study
CA-IF063	*sod5Δ/Δ*	SC5314, *sod5Δ::*FRT/*sod5Δ::*FRT	This study
CA-IF067	*sod5Δ/Δ sod4Δ/Δ sod6Δ*	SN152, *sod5Δ::*cm*LEU1/sod5Δ::Cd HIS1 sod4Δ::*FRT/*sod4Δ::*FRT *sod6Δ::*FRT/*SOD6*	This study
CA-IF070	*sod5Δ/Δ sod4Δ/Δ sod6Δ/Δ*	SN152, *sod5Δ::*cm*LEU1/sod5Δ::Cd HIS1 sod4Δ::*FRT/*sod4Δ::*FRT *sod6Δ::*FRT/*sod6Δ::*FRT	This study
